# Stepwise Diagnostic Evaluation of Chinese Large Language Models: Comparative Study of Common and Rare Diseases

**DOI:** 10.2196/89963

**Published:** 2026-08-06

**Authors:** Jiayi Wang, Jiao Yang, Rui Guo

**Affiliations:** 1 Department of Health Management and Policy, School of Public Health, Capital Medical University, No. 10 Xitoutiao, Youanmenwai, Fengtai District, Beijing, Beijing, 100069, China, 86 010 83911303

**Keywords:** large language models, clinical decision-making, diagnostic accuracy, decision support systems, artificial intelligence

## Abstract

**Background:**

Large language models (LLMs) are increasingly applied in clinical decision support, yet their diagnostic performance in Chinese-language settings and under realistic clinical workflows remains unclear. In particular, how LLMs perform across diseases with different prevalence and under stepwise diagnostic processes has not been well characterized.

**Objective:**

This study aimed to evaluate the diagnostic capabilities of LLMs for common diseases and rare diseases using clinical vignettes within a hypothetico-deductive framework and to identify their potential and limitations for clinical diagnosis.

**Methods:**

We evaluated 4 Chinese LLMs (Doubao 1.5, DeepSeek-V3, Kimi K1.5, and Leftdoctor GPT 3.5) using 56 clinical cases (28 chronic obstructive pulmonary disease [COPD], and 28 relapsing polychondritis [RP]) sourced from the China Clinical Case Results Database (March 31-April 14, 2025). Patient information was provided incrementally, starting with the initial medical history, followed by physical examination, and laboratory results. Evaluation metrics included top-3 accuracy (RTop3D), top-1 accuracy (RTopD), final diagnostic accuracy (RFA), and mean reciprocal rank (MRR). Statistical analysis was performed using generalized estimating equations (GEE), Friedman tests, and Wilcoxon signed-rank tests with Bonferroni correction. In addition, a qualitative analysis was conducted to characterize recurrent patterns of diagnostic errors.

**Results:**

LLMs demonstrated significantly higher diagnostic accuracy for COPD compared to RP across all metrics (*P*<.001). Diagnostic accuracy improved after additional clinical information was provided, with the improvement mainly observed in RP cases. In RP, diagnostic accuracy increased from 32.14% to 71.43% for DeepSeek and from 35.71% to 78.57% for Doubao, whereas COPD accuracy remained consistently high across all diagnostic stages (82.14%‐92.86%). For COPD, ranking performance was high and comparable among all models (MRR range: 0.82‐0.89; *P*=.71). In RP, diagnostic performance differed significantly among models (MRR range: 0.10‐0.39; *P*<.001). Qualitative analysis showed that COPD errors were mainly related to a failure to recognize specific features, whereas RP errors involved more diverse patterns, particularly the neglect of negative evidence and the failure to recognize specific features.

**Conclusions:**

Chinese LLMs demonstrated relatively strong diagnostic performance for common diseases such as COPD, but lower and less stable performance for rare diseases such as RP. Additional clinical information improved diagnostic accuracy primarily in RP cases, although differences between models remained evident under diagnostically complex conditions. Error patterns in RP cases suggest that current LLMs remain limited in their ability to integrate complex clinical information and exclusionary findings. Careful evaluation and appropriate clinical oversight remain important for their application in clinical practice.

## Introduction

Recent advances in large language models (LLMs) have demonstrated their transformative potential across diverse health care domains. These applications range from automating medical documentation and patient education to providing personalized health consultation and drug discovery [1-5]. Despite this broad use, AI-driven diagnostic assistance remains one of the most promising and impactful scenarios in clinical practice [6-9]. Given the critical and complex nature of clinical decision-making, exceptionally high accuracy is required. However, diagnostic reasoning in real-world practice often involves uncertainty, incomplete information, and iterative hypothesis refinement [10]. In this context, the application of LLMs raises concerns not only about “hallucinations” but also about their reliability and stability in supporting complex clinical reasoning processes [11]. Therefore, their diagnostic performance must be carefully and systematically evaluated before being considered for clinical use.

China’s LLM development is progressing rapidly with strong national support. However, most existing studies focus on evaluating LLMs’ diagnostic capabilities in English [12-16]. Given that some research indicates ChatGPT performs better with English input compared to Chinese, and that Chinese LLMs leverage large Chinese corpora and training data representative of the Chinese population, evaluating their diagnostic ability in the Chinese context is essential for their application in Chinese clinical settings. This necessity is further underscored by the unique challenges of Chinese medical natural language processing (NLP), where models must navigate highly unstructured clinical notes characterized by complex syntactic structures and nonstandardized medical abbreviations. Although some studies have explored Chinese LLMs’ diagnostic ability in Chinese contexts, research in this area remains limited [17].

Early assessments of LLMs’ diagnostic competence often used multiple choice questions (MCQs), which may not accurately reflect real-world clinical performance due to their structured nature. Recent studies have shifted toward clinical vignette-based evaluations that better mimic actual clinical scenarios [18-22]. Most of these evaluations provide LLMs with complete patient data simultaneously. However, real clinical reasoning typically follows a hypothetico-deductive process, where clinicians generate an initial differential diagnosis (DDx) from limited information, then iteratively refine these hypotheses with additional data. Assessment methods simulating this incremental information provision have demonstrated reduced diagnostic accuracy in LLMs compared to approaches presenting all data at once.

The diagnostic ability of LLMs is also highly dependent on specific disease domains and task types. While previous studies have explored LLMs’ diagnostic abilities for common and rare diseases, most have been limited to single disease categories, and comparative analyses across multiple disease types are scarce. Consequently, the understanding of the variability in LLMs’ diagnostic performance across diverse disease groups remains limited [23-27].

In addition to the limitations discussed above, a further methodological gap exists in the current literature. Most prior studies evaluating the diagnostic performance of LLMs rely on various forms of model adaptation, including prompt engineering, few-shot learning, or task-specific fine-tuning [28,29]. While these approaches may enhance performance, they confound the assessment of the model’s intrinsic diagnostic capability, making it difficult to disentangle the contribution of the underlying model from that of external optimization strategies. Establishing the baseline performance of unmodified foundation models is particularly important in the context of health care. Before LLMs can be deployed in real-world clinical settings, models intended for health care implementation must be demonstrated to be accurate, reliable, and safe for use in patient care. Without a clear understanding of their intrinsic capabilities under minimal intervention, it is challenging to determine whether observed performance reflects genuine reasoning ability or artifacts of task-specific optimization.

In summary, this study used a step-by-step approach based on the hypothetico-deductive model to comparatively assess the zero-shot diagnostic reasoning capabilities of Chinese LLMs for common and rare diseases in simulated real clinical scenarios. All models were evaluated in their original form under consistent conditions, allowing for a more direct comparison of their diagnostic performance. This design helps to provide a clearer view of how LLMs perform in clinically relevant settings and may offer useful insights for their potential application in practice.

## Methods

### Overview

This study was conducted and reported in accordance with the Standards for Reporting Diagnostic Accuracy Studies (STARD) guidelines to ensure transparent and complete reporting of diagnostic accuracy.

### Selection of Disease Types

This study selected representative disease models to evaluate LLM performance across different diagnostic reasoning profiles.

Chronic obstructive pulmonary disease (COPD) was selected to represent common diseases due to its significant public health burden and high incidence. COPD is a highly prevalent internal medicine condition, affecting approximately 380 million people globally and causing over 3 million deaths annually, making it the third leading cause of mortality worldwide. In China, the overall prevalence of COPD among individuals aged 40 and above is 8.6% [30]. Beyond its prevalence, COPD follows well-defined diagnostic criteria, making it a suitable model for assessing LLMs’ ability to recognize high-frequency clinical patterns.

Relapsing polychondritis (RP) was selected to represent rare diseases because of its high disability rate, frequent diagnostic delays, and misdiagnoses in clinical practice, which place a substantial economic burden on both patients and society. RP is an autoimmune disease with an unclear pathological mechanism, affecting multiple organ systems. Consequently, RP was selected as our rare disease case due to these clinical challenges and its significant impact on patients. RP was specifically chosen because its diagnosis requires synthesizing heterogeneous clinical signals across multiple organ systems, making it an appropriate model for testing models’ capacity for multisystem information integration rather than simple pattern matching [31].

The selection of these 2 diseases allowed for a controlled comparison between localized, pattern-consistent conditions and systemic, complex scenarios. Although COPD and RP involve different physiological systems, all cases were developed using a consistent clinical framework, including the chief complaint, history of present illness, physical examination, and key investigations. This approach was intended to keep the diagnostic process comparable across cases by focusing on how clinical information is integrated during reasoning.

The models evaluated in this study are general-purpose LLMs trained on diverse medical corpora rather than specialty-specific datasets. Evaluating their performance across diseases with distinct diagnostic structures, therefore, reflects their baseline reasoning capabilities and reduces the likelihood that results are driven by domain-specific familiarity.

### Case Development

We sourced cases from the China Clinical Case Results Database (CMCR), a national large-scale clinical case results publishing platform funded by the Chinese Association for Science and Technology and constructed by the Journal of the Chinese Medical Association. The database contains peer-reviewed clinical cases of high professional standard (eg, COPD diagnosed per Global Initiative for Chronic Obstructive Lung Disease [GOLD] criteria and RP per McAdam criteria) and provides established reference diagnoses.

**Figure 1.**
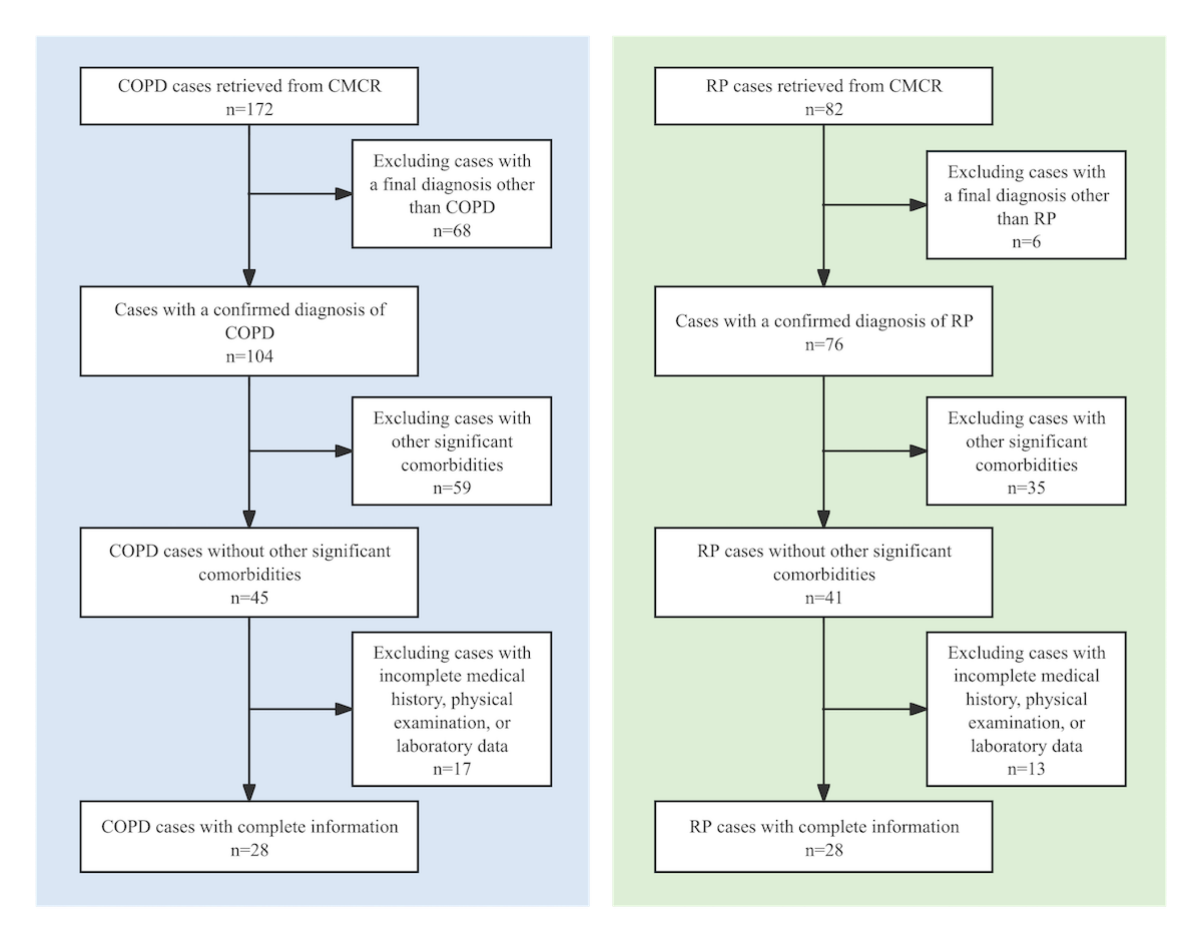
Case selection flowchart. CMCR: China Clinical Case Results Database; COPD: chronic obstructive pulmonary disease; RP: relapsing polychondritis.

Cases of COPD and RP were retrieved between March 31 and April 14, 2025. To ensure suitability for evaluating LLM’s diagnostic performance, cases were selected based on the clarity and clinical consistency of their primary diagnoses. The final diagnosis was required to be either COPD or RP, and cases had to include detailed medical history, physical examination, laboratory tests, and other supporting diagnostic information. Cases with significant comorbidities that could confound diagnostic interpretation were excluded (Figure 1).

To leverage the advantages of LLMs in processing unstructured data, we specifically adapted these clinician-written summaries into a format approximating the authentic language and descriptive style of real-world patients. This involved omitting nonessential sections (eg, titles, treatment discussions, and accompanying tables and videos) and naturalizing the language. Specifically, overly specialized medical expressions were modified to approximate natural patient language, as real-world patients seldom use the professional jargon found in standardized databases (Figure S1 in Multimedia Appendix 1). In addition, this adaptation process also aimed to reduce the likelihood that cases could be directly recognized or matched to memorized training data, thereby mitigating potential bias related to data contamination.

This prespecified and well-defined reference standard reduced potential diagnostic ambiguity during the evaluation phase and enabled consistent comparison of LLM-generated outputs against the ground truth. It also provided a structured basis for applying a rule-based semantic matching approach in the outcome assessment. Furthermore, this standardized case construction ensured that diagnostic reasoning was primarily driven by structured clinical information rather than domain-specific knowledge alone, thereby mitigating potential confounding arising from differences in medical specialties.

As this study was based on retrospective clinical vignettes and did not involve direct patient intervention or participant recruitment, no adverse events occurred during the research process. Baseline demographic characteristics, including patient age and sex, were extracted alongside the clinical information for each case.

### LLM Selection

We selected 4 Chinese LLMs for evaluation, including 3 general LLMs and one health care-specific LLM. All LLMs are open-source and independently developed in China. The general LLMs selected were Doubao (Doubao 1.5), DeepSeek (DeepSeek-V3), and Kimi (Kimi K1.5), which ranked as the top 3 AI products in terms of Chinese active users in February 2025. The health care–specific model, Leftdoctor GPT (Leftdoctor GPT 3.5), was selected from the “2024 China Healthcare LLMs Top 30” list released on November 5, 2024, by the Chinese Academy of Sciences ('China Internet Week’) and the Center for Informatization Study. All models were accessed through their publicly available interfaces and evaluated under consistent conditions without task-specific adaptation. Models designed for specialized applications, including patient services, medical image analysis, scientific research and innovation, hospital management, and medical record writing, were excluded to ensure comparability across general diagnostic tasks.

### LLM Prompts

**Figure 2.**
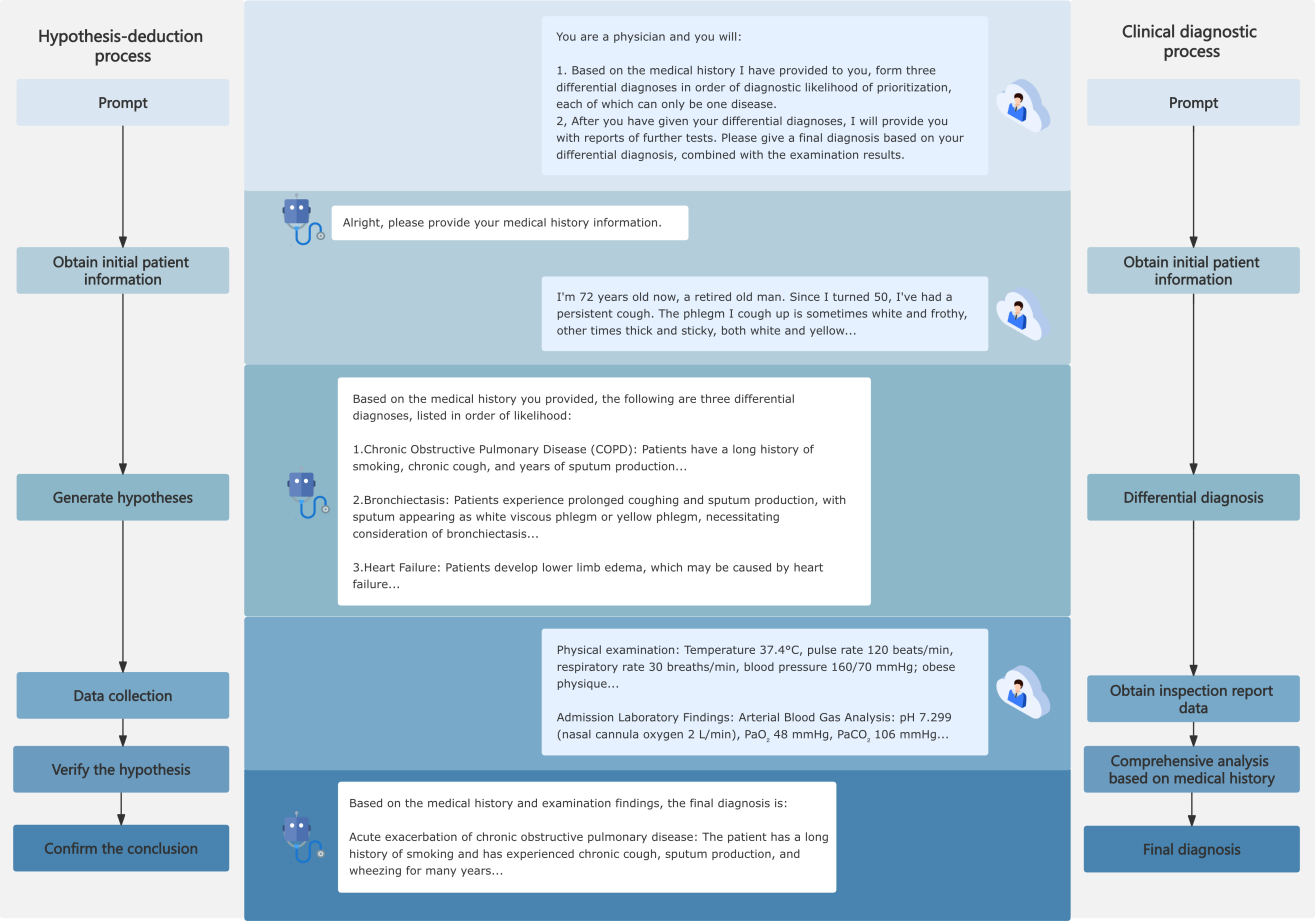
Clinical scenario interaction examples of simulated human-computer interaction under clinical diagnosis and hypothesis deduction processes. COPD: chronic obstructive pulmonary disease.

To better simulate clinical practice, we provided task prompts to LLMs, requiring them to generate a differential diagnosis based on initial information and then provide a final diagnosis after receiving examination results (Figure 2). As the study was conducted using Chinese LLMs, the original prompts were administered in Chinese (the complete Chinese versions are provided in Multimedia Appendix 1). The English translations of the prompts were:

You are a physician and you will:

1. Based on the medical history I have provided to you, form 3 differential diagnoses in order of diagnostic likelihood of prioritization, each of which can only be one disease.
2. After you have given your differential diagnoses, I will provide you with reports of further tests. Please give a final diagnosis based on your differential diagnosis, combined with the examination results.

### Evaluation Protocol

The evaluation was framed by the deterministic reference diagnoses retrieved from the CMCR database. Since these gold-standard diagnoses were prespecified and verified, the assessment was implemented as a rule-based semantic matching protocol. To maintain consistency, we established predefined synonym boundaries referencing standardized medical vocabularies, such as *ICD-10 (International Classification of Diseases, Tenth Revision) *or MeSH, in alignment with established medical AI benchmarks. Within this framework, a model-generated diagnosis was recorded as correct if it aligned with the ground truth or its recognized clinical synonyms. The assessment was performed independently by 2 researchers (JYW and XNL) who were blinded to the identity of the LLMs generating the responses, with any discrepancies adjudicated by a third senior evaluator (JY) to reach a final consensus. This structured scoring process yielded a high level of interrater agreement (Cohen kappa=.950).

### Outcome Indicators

The diagnostic performance of the LLMs was evaluated using three primary outcome indicators: the rate of final diagnosis within the top 3 DDx list (RTop3D), the rate of final diagnosis as top diagnosis (RTopD), and the rate of final diagnostic accuracy (RFA). RTopD and RTop3D were used to assess the accuracy of the models’ initial diagnostic hypotheses, reflecting their diagnostic breadth and the reliability of the generated candidate list. RFA was defined as the accuracy of the final decision produced after the model integrated supplementary clinical information. By analyzing the transition from initial differential accuracy (RTopD and RTop3D) to RFA, we assessed the models’ clinical reasoning and their capacity to refine diagnostic conclusions as the case progressed. Detailed definitions, assignment standards, and calculation formulas for these metrics are provided in Table S1 in Multimedia Appendix 1.

The mean reciprocal rank (MRR) was used to evaluate the precision of the LLMs in ranking the correct diagnosis. The reciprocal rank for each case was determined by the position of the gold-standard diagnosis among the top 3 differential diagnoses provided by the model. The MRR was calculated using the following formula:


$$MRR=\frac{1}{C}\sum_{i=1}^{C}\frac{1}{r_{i}}$$

where C corresponds to the number of cases on which the metric is evaluated, and *r*_*i*_ is the rank of the first occurrence of a correct answer in the final list for case *i*. Specifically, a score of 1, .5, or .33 was assigned if the correct diagnosis appeared as the first, second, or third suggestion, respectively. In any case where the correct diagnosis is ranked beyond the top 3 (*r*_*i*_>3) or is absent from the list, the contribution to the MRR is set to 0. This metric effectively rewards models that consistently place the gold-standard diagnosis at higher positions within the differential diagnosis list.

### Qualitative Error Analysis

To characterize recurrent patterns of diagnostic errors generated by the LLMs, a qualitative analysis was conducted on all cases in which the final diagnosis was incorrect. Following an inductive thematic coding process, 2 independent raters (JYW and XNL) reviewed the reasoning trajectories and iteratively identified five recurrent error domains: (1) failure to recognize specific features, involving omission of critical diagnostic clues or pathognomonic findings explicitly provided in the case description; (2) incorrect attribution, where clinical findings were identified but misinterpreted or assigned to an incorrect etiology; (3) failure in multisystem information integration, characterized by the inability to synthesize manifestations across multiple organ systems into a coherent systemic diagnosis; (4) frequency-based matching bias, referring to the tendency to prioritize high-prevalence diseases despite recognizing disease-specific clues suggestive of a rare condition; and (5) neglect of negative evidence, defined as the failure to incorporate exclusionary findings (eg, negative laboratory results) into differential diagnostic reasoning.

A multilabel coding approach was adopted, allowing a single diagnostic error to be assigned to multiple domains when applicable. Discrepancies between the primary raters were resolved by a third senior evaluator (JY). Given the nonmutually exclusive nature of these error categories, interrater reliability was assessed by decomposing the coding task into 5 independent binary classification problems. The category-specific Cohen kappa coefficients ranged from .824 to .939, with an average value of .898, indicating strong agreement in qualitative categorization.

### Quality Control

Several measures were adopted to enhance assessment reliability. Before formal evaluation, the 2 primary raters (JYW and XNL) conducted a calibration session using a representative subset of cases to align interpretations of the scoring criteria and the 5 predefined error domains. All assessments were performed in independent dialogue sessions using previously unused accounts to minimize potential information carryover between cases, and the raters were blinded to the identity of the LLMs generating the outputs, which were anonymized prior to evaluation. Residual disagreements were resolved through blinded adjudication by a third senior evaluator (JY). In addition, screenshots of all model outputs were archived to ensure traceability and allow subsequent verification of the extracted data.

### Statistical Analysis

Data were entered and organized in Excel, and statistical analyses were conducted using R software (version 4.6.0; R Foundation for Statistical Computing). Diagnostic accuracy was summarized as counts and percentages. There was no missing data in this study, as all 56 clinical vignettes were complete and all 4 LLMs successfully generated responses for each assigned case. Furthermore, no indeterminate or uninterpretable model outputs were encountered; all LLM responses were determinate and evaluable according to the predefined scoring criteria.

To account for within-case correlation, where each clinical vignette was evaluated by 4 LLMs across 2 diagnostic stages, yielding 448 individual diagnostic responses (the analytic unit), generalized estimating equations (GEE) with a binary logistic link and an exchangeable working correlation structure were fitted using the *geepack package* in R. An exchangeable structure was chosen because repeated observations within each case lacked a natural temporal order.

The GEE analyses proceeded in 2 stages. First, separate models were constructed for each primary endpoint (RTopD, RTop3D, and RFA). These models included LLM type and disease group (COPD vs RP) as main effects, along with their interaction (LLM × disease), to evaluate performance differences across disease categories. Second, to examine diagnostic refinement, a combined GEE model was fitted by pooling initial and final assessments and adding diagnostic stage as a within-subject factor. This model included the relevant main effects and key interactions (stage × LLM and stage × disease) to quantify changes in diagnostic accuracy. For all GEE models, results are reported as odds ratios (ORs) with 95% CIs, and pairwise comparisons of estimated marginal means were performed using Bonferroni adjustment. To assess the robustness of the primary statistical inferences, sensitivity analyses were conducted by comparing the primary exchangeable working correlation structure with an alternative independent working correlation structure. Consistency of parameter estimates, odds ratios, CIs, and statistical inferences across correlation specifications was examined to evaluate the robustness of the findings to the choice of working correlation structure.

To evaluate diagnostic ranking performance, the MRR was computed for each model as a descriptive summary metric. Given the repeated-measures design, in which the same clinical cases were evaluated across the 4 LLMs, and the nonnormal distribution of the ranking data, statistical comparisons were conducted using the Friedman test based on case-level reciprocal rank scores. When a statistically significant overall difference was detected, post-hoc pairwise comparisons were conducted using the Wilcoxon signed-rank test. To control the family-wise error rate across the 6 possible LLM pairs, the Bonferroni correction was applied (adjusted significance threshold: .008).

A 2-sided *P*<.05 was considered statistically significant.

### Ethical Considerations

This study was conducted in accordance with the protocol approved by the Ethics Committee of Capital Medical University (approval number: 2025SY-166) and adhered to the ethical principles of the Declaration of Helsinki. The research used standardized clinical vignettes derived from a retrospective database for the purpose of benchmarking AI models. In alignment with institutional ethical guidelines for the use of deidentified, retrospective data, all patient information was fully anonymized prior to analysis to ensure that no individuals could be identified. No direct intervention or interaction with human participants occurred during this study. All research procedures were performed strictly following the data privacy and confidentiality standards approved by the institutional review board.

## Results

### Baseline Characteristics of Clinical Cases

A total of 56 clinical vignettes were evaluated in this study, comprising 28 COPD cases and 28 RP cases. Across all cases, the mean age of the patients was 57.6 (SD 18.7) years, with a sex distribution of 43 males (76.8%) and 13 females (23.2%). In the COPD cohort, the mean age was 69.1 (SD 11.3) years (24 males and 4 females), while in the RP cohort, the mean age was 46.2 (SD 17.8) years (19 males and 9 females).

### Diagnostic Accuracy by LLMs

Across the 3 diagnostic performance metrics, differences in diagnostic accuracy were observed among the LLMs. Compared with Leftdoctor GPT, both DeepSeek and Doubao consistently showed higher diagnostic accuracy across all 3 metrics (RTop3D: OR=4.50, 95% CI 1.71-11.83, *P*=.002; RTopD: OR=6.16 and OR=7.22, 95% CI 1.62-23.36 and 1.86-28.03, *P*=.008 and *P*=.004 for DeepSeek and Doubao, respectively; final diagnosis: OR=6.25 and 9.17, 95% CI 2.55-15.34 and 3.38-24.90 for DeepSeek and Doubao, respectively, all *P*<.001). In contrast, KIMI did not differ significantly from Leftdoctor GPT for any of the metrics (all *P*>.05). Overall, DeepSeek and Doubao demonstrated better diagnostic performance, whereas the performance of KIMI was comparable to that of Leftdoctor GPT (Table 1).

**Table 1.** Generalized estimating equations analysis of diagnostic accuracy by large language model, disease, and diagnostic stage. Odds ratio with 95% CIs were estimated using generalized estimating equations with an exchangeable working correlation structure to account for repeated measurements within the same cases. The model included main effects of large language model, disease group, and diagnostic stage, as well as interaction terms (large language model × disease, disease × stage, and large language model × stage). Reference categories are indicated in the “Reference” column. Odds ratios greater than 1 indicate higher odds of correct diagnosis compared with the reference group. All *P* values are two-sided.

Variable, metric, and comparison	Reference	OR^a^ (95% CI)	*P* values
LLM^b^	Leftdoctor GPT		
RTop3D^c^			
DeepSeek		4.50 (1.71-11.83)	.002
Doubao		4.50 (1.71-11.83)	.002
KIMI		1.30 (0.53-3.21)	.56
RTopD^d^			
DeepSeek		6.16 (1.62-23.36)	.008
Doubao		7.22 (1.86-28.03)	.004
KIMI		1.56 (0.66-3.70)	.31
RFA^e^			
DeepSeek		6.25 (2.55-15.34)	<.001
Doubao		9.17 (3.38-24.90)	<.001
KIMI		0.83 (0.45-1.54)	.56
Disease	RP		
RTop3D			
COPD^f^		50 (10.11-247.23)	<.001
RTopD			
COPD		108.33 (16.67-703.98)	<.001
RFA^g^			
COPD		11.50 (3.24-40.86)	<.001
Stage			
No metric			
Top-1 Differential Diagnosis Stage	Final Assessment Stage	0.307 (0.09-1.02)	.05
No metric	Leftdoctor GPT × Final Assessment Stage		
DeepSeek × Top-1 Differential Diagnosis Stage		0.60 (0.20-1.85)	.38
Doubao× Top-1 Differential Diagnosis Stage		0.49 (0.18-1.34)	.16
KIMI × Top-1 Differential Diagnosis Stage		0.87 (0.35-2.16)	.77
No metric			
COPD × Top-1 Differential Diagnosis Stage	RP× Final Assessment Stage	4.10 (0.88-19.11)	.07
Interaction terms	Leftdoctor GPT × RP		
RTop3D			
Doubao × COPD		0.12 (0.03-0.56)	.006
DeepSeek × COPD		0.22 (0.04-1.28)	.09
KIMI × COPD		0.77 (0.34-4.16)	.70
RTopD			
Doubao × COPD		0.08 (0.01-0.46)	.005
DeepSeek × COPD		0.16 (0.02-1.18)	.07
KIMI × COPD		0.64 (0.17-2.47)	.52
RFA			
Doubao × COPD		0.20 (0.06-0.71)	.01
DeepSeek × COPD		0.45 (0.08-2.68)	.38
KIMI × COPD		3.39 (0.91-12.63)	.07

a OR: odds ratio.

b LLM: large language model.

c RTop3D: proportion of cases in which the correct diagnosis was ranked within the top three differential diagnoses.

d RTopD: proportion of cases in which the correct diagnosis was ranked first.

e RFA: proportion of cases in which the final diagnosis was correctly identified.

f COPD: chronic obstructive pulmonary disease.

g RP: relapsing polychondritis.

### Diagnostic Accuracy by Disease

Significant differences in diagnostic accuracy were observed between the 2 diseases across all 3 metrics. Compared with RP, diagnostic accuracy was substantially higher for COPD for RTop3D (OR=50; 95% CI 10.11 -247.23; *P*<.001), RTopD (OR=108.33; 95% CI 16.67 -703.98; *P*<.001), and RFA (OR=11.50; 95% CI 3.24 -40.86*; P*<.001). These findings indicate that the LLMs achieved markedly better diagnostic performance for COPD cases than for RP cases (Table 1).

### Changes in Diagnostic Accuracy With Additional Clinical Information

The incorporation of additional clinical information led to an overall upward trend in diagnostic accuracy, although the difference between the top-1 differential diagnosis and final assessment stages was of borderline statistical significance (*P*=.05) (Table 1). Furthermore, no significant interaction was observed between diagnostic stage and LLM type (all *P*>.05), indicating that the models responded similarly to the supplementary data.

Despite the lack of a strictly significant interaction between diagnostic stage and disease type (*P*=.07), descriptive results revealed distinct performance trajectories between the 2 disease categories (Table 2). For COPD, all LLMs already achieved high diagnostic accuracy early in the top-1 differential diagnosis stage (82%‐93%), with minimal further improvement after additional clinical information was supplied. In contrast, diagnostic accuracy for RP was substantially lower at the top-1 differential diagnosis stage (7%‐36%) but improved markedly by the final assessment stage (25%‐79%). These findings suggest that the overall improvement across diagnostic stages was primarily driven by marked gains in RP accuracy, whereas diagnostic performance for COPD remained consistently high throughout the diagnostic process.

**Table 2.** Accuracy comparison of LLMs^a^ in common and rare diseases. Values are presented as n (%), with percentages calculated out of 28 cases per disease.

Metric	DeepSeek, n (%)	KIMI, n (%)	Doubao, n (%)	Leftdoctor GPT, n (%)
COPD^b^				
RTop3D^c^	25 (89.29)	25 (89.29)	23 (82.14)	25 (89.29)
RTopD^d^	25 (89.29)	25 (89.29)	23 (82.14)	25 (89.29)
RFA^e^	26 (92.86)	26 (92.86)	25 (89.29)	23 (82.14)
RP^f^				
RTop3D	12 (42.86)	5 (17.86)	12 (42.86)	4 (14.29)
RTopD	9 (32.14)	3 (10.71)	10 (35.71)	2 (7.14)
RFA	20 (71.43)	7 (25.00)	22 (78.57)	8 (28.57)

a LLM: large language model.

b COPD: chronic obstructive pulmonary disease.

c RTop3D=proportion of cases in which the correct diagnosis was ranked within the top three differential diagnoses.

d RTopD=proportion of cases in which the correct diagnosis was ranked first.

e RFA=proportion of cases in which the final diagnosis was correctly identified.

f RP: relapsing polychondritis.

### Interaction Between Disease Type and Model

A significant interaction between disease type and LLM was observed for all diagnostic metrics, including RTop3D (Wald χ²_3_=9.42; *P*=.02), RTopD (Wald χ²_3_=8.52; *P*=.04), and RFA (Wald χ²_3_=18.62; *P*<.001), indicating that differences in LLM performance varied across disease.

Parameter estimates from the GEE model indicated that this interaction was mainly associated with Doubao. Specifically, the interaction between Doubao and COPD was statistically significant across all three diagnostic metrics: the RTop3D metric (OR=.12, 95% CI .03-.56; *P*=.006) and the RTopD metric (OR=.08, 95% CI .01-.46; *P*=.005), as well as for the RFA metric (OR=.20, 95% CI .06-.71; *P*=.01). In contrast, the interaction terms for DeepSeek and KIMI were not statistically significant (all *P*>.05).

These findings suggest that the variation in diagnostic performance across diseases was mainly attributable to disease-specific performance differences in the Doubao model.

### LLM Differential Diagnosis Ranking Performance (MRR)

Ranking performance, which accounts for both the correctness and rank position of differential diagnoses, was evaluated using MRR.

For COPD diagnosis, DeepSeek, KIMI, and Leftdoctor GPT achieved the highest MRR scores (.89 (SD .32)), followed by Doubao (mean .82, SD .39). Given the repeated-measures design of the evaluation across the same clinical cases, the Friedman test was used. The analysis indicated no statistically significant difference in diagnostic ranking performance among the 4 LLMs (χ²_3_=1.385; *P*=.71; Table S2 in Multimedia Appendix 1).

**Figure 3.**
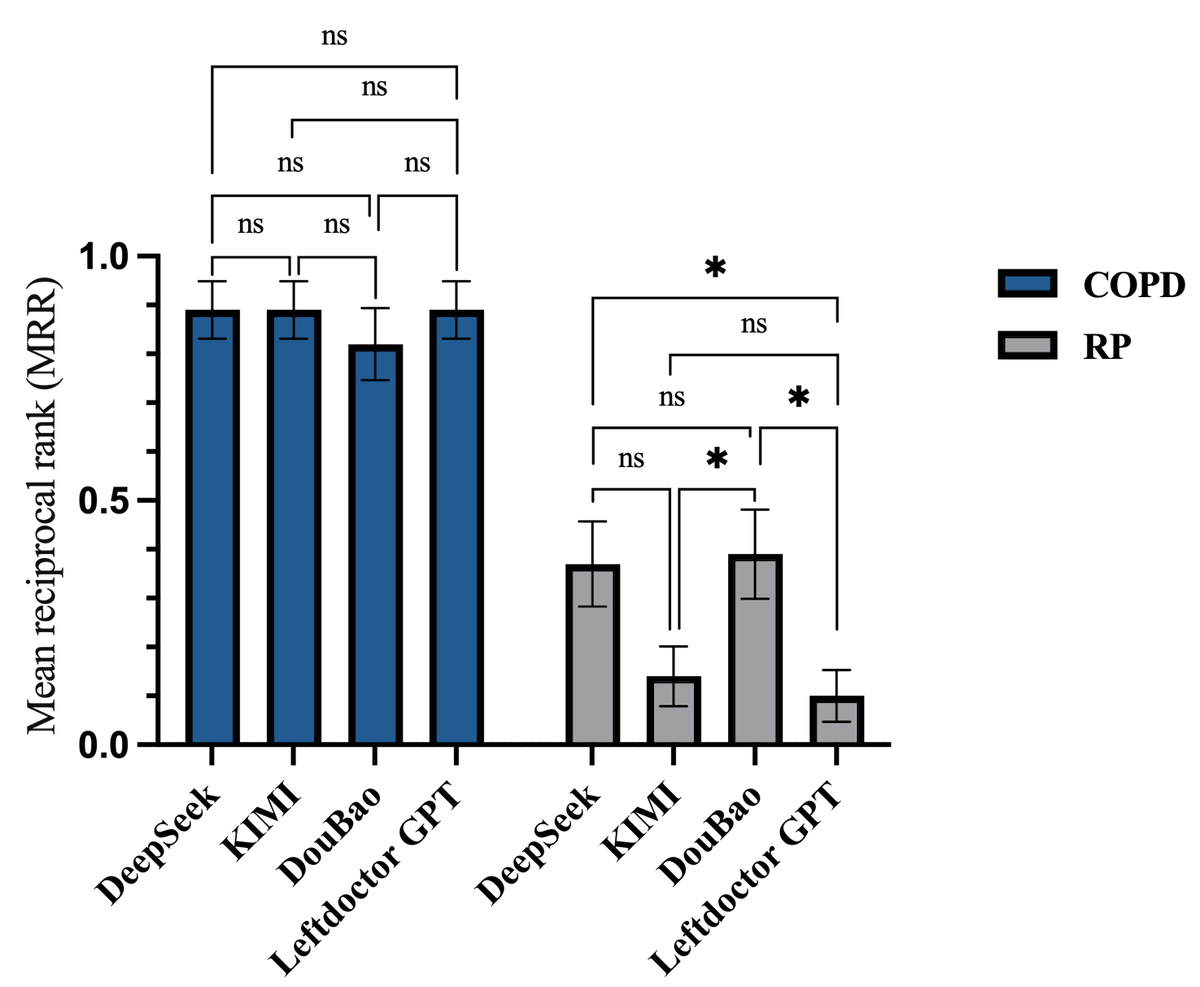
Mean reciprocal rank across large language models for chronic obstructive pulmonary disease and relapsing polychondritis. Bars represent mean and standard error of the mean (n=28 cases per group). Global comparisons among the four models were performed using the Friedman test based on case-level performance scores. Post hoc pairwise comparisons were conducted via the Wilcoxon signed-rank test, also performed at the case level, with Bonferroni correction for multiple testing. Asterisks (*) indicate statistically significant differences (*P*<.05); ns indicates no significant difference (*P*>.05). SEM: standard error of the mean; MRR: mean reciprocal rank; LLMs: large language models; COPD: chronic obstructive pulmonary disease; RP: relapsing polychondritis.

For RP diagnosis, diagnostic ranking performance differed notably across the LLMs. Doubao achieved the highest MRR score (.39, SD .48), followed by DeepSeek (.37, SD .46), KIMI (.14, SD .32), and Leftdoctor GPT (.10, SD .28). Given the repeated-measures design, a Friedman test was conducted based on case-level performance scores, revealing a statistically significant overall difference among the models (χ²_3_=21.933; *P*<.001). Post-hoc pairwise comparisons using the Wilcoxon signed-rank test, also performed at the case level, with Bonferroni correction (adjusted significance threshold of *α*=.0083 for 6 comparisons), indicated that both Doubao and DeepSeek significantly outperformed Leftdoctor GPT (both unadjusted *P*=.002). Furthermore, Doubao significantly outperformed KIMI (unadjusted *P*=.008). Although DeepSeek scored higher than KIMI, this difference did not reach the adjusted significance threshold after Bonferroni correction (unadjusted *P*=.01). Differences between all other LLM pairs were not statistically significant (Figure 3, Table S3 in Multimedia Appendix 1).

### Qualitative Analysis of Diagnostic Errors

Distinct error patterns were observed between COPD and RP cases (Table 3). Among the 12 incorrect COPD diagnoses, errors were limited to failure to recognize specific features (10, 83.3%) and incorrect attribution (2, 16.7%), with no concurrent error types identified.

Among the 55 incorrect RP diagnoses, a total of 61 error events were identified, with 6 cases (10.9%) involving multiple error types. The most common errors were neglect of negative evidence (21, 34.4%) and failure to recognize specific features (14, 23%), followed by incorrect attribution (9, 14.8%), frequency-based matching bias (9, 14.8%), and failure in multisystem information integration (8, 13.1%).

Model-specific differences were also observed. Leftdoctor GPT showed the highest number of errors (21), most commonly neglect of negative evidence (10). Kimi demonstrated the broadest distribution of error categories (21), whereas DeepSeek errors were mainly related to neglect of negative evidence and failure to recognize specific features (5 each). Doubao showed the fewest errors (6), without a dominant error category.

Sensitivity analyses were conducted using alternative independent working correlation structures. The resulting parameter estimates, ORs, CIs, and statistical inferences were materially consistent with those obtained under the primary exchangeable structure, indicating that the study findings were robust to the choice of working correlation structure (Multimedia Appendix 2).

**Table 3.** Distribution of diagnostic error domains across four LLMs for COPD and RP cases.

Error domain	DeepSeek	Kimi	Doubao	Leftdoctor GPT	Total, n (%)
COPD^a^ (n=12 error events)					
Failure to recognize specific features	2	2	2	4	10 (83.3)
Incorrect attribution	0	0	1	1	2 (16.7)
RP^b^ (n=61 error events)					
Neglect of negative evidence	5	5	1	10	21 (34.4)
Failure to recognize specific features	5	5	2	2	14 (23)
Incorrect attribution	1	4	1	3	9 (14.8)
Frequency-based matching bias	0	4	1	4	9 (14.8)
Failure in multisystem information integration	2	3	1	2	8 (13.1)

a COPD: chronic obstructive pulmonary disease.

b RP: relapsing polychondritis.

## Discussion

### Principal Findings

This study evaluated the diagnostic performance of 4 Chinese LLMs across common and rare diseases using a stepwise, hypothetico-deductive framework. Three main findings emerged. First, all models demonstrated substantially higher diagnostic accuracy for the common disease (COPD) compared with the rare disease (RP). Second, incremental clinical information improved diagnostic accuracy primarily in rare disease scenarios, with considerable variation across models. Third, differences between models were minimal under relatively straightforward diagnostic conditions but became more pronounced in diagnostically challenging cases.

### Diagnostic Performance Disparity Between Common and Rare Diseases

A prominent finding of this study is the consistent performance gap between common and rare diseases across all evaluated models. Similar patterns have been reported in recent studies, where LLM performance declines in complex or rare disease scenarios [32]. While this phenomenon is often attributed to differences in data availability [33,34], our results suggest that it may also relate to the distinct clinical structures of these conditions and the influence of learned associations on model outputs.

For common diseases such as COPD, clinical presentations tend to align with frequently encountered and well-represented patterns in training data. Qualitative observations show that errors in the COPD group were largely confined to the failure to identify specific features, while instances of incorrect attribution were infrequent across multiple evaluations. This suggests that when key diagnostic information is correctly recognized, models can generally map it to the intended diagnosis. In these situations, LLMs can rely on strong probabilistic associations to produce accurate and stable diagnostic suggestions [35].

The disparity between COPD and RP extends to differences in organ system involvement and diagnostic breadth. While COPD is primarily localized to the respiratory system, RP is a systemic condition characterized by multiorgan involvement and a complex symptom profile [25]. This complexity requires the synthesis of heterogeneous signals, a task where LLMs showed limitations. Our analysis identified a higher diversity of error types in RP cases, including the neglect of negative evidence and failures in multisystem integration. The presence of frequency-based matching bias, where models prioritized high-prevalence conditions despite identifying RP-specific cues, indicates that a reliance on learned associations may affect the rigorous refinement required for complex, multisystemic conditions [36].

Consequently, their performance remains less stable in low-prevalence or diagnostically complex scenarios, which has important implications for their safe clinical application [37,38].

### The Role of Incremental Information in Diagnostic Refinement

This study also provides insight into how LLMs respond to sequentially provided clinical information. While additional data had a limited impact on diagnostic accuracy for COPD, it significantly improved performance in RP cases for some models. This asymmetry suggests that the use of incremental information depends on the level of initial diagnostic certainty [39]. In the COPD group, the high initial diagnostic accuracy across models left limited room for further improvement through incremental data. Although infrequent diagnostic errors occurred in the final assessment stage, such as the omission of specific features in either the history or laboratory results, the strong initial signals associated with common conditions allowed LLMs to maintain stable performance, largely unaffected by the addition of new information.

In contrast, when initial diagnostic signals are weak or ambiguous, as in rare diseases, the introduction of new information can meaningfully shift the probability distribution and improve diagnostic accuracy [40]. However, the qualitative observations indicate that while incremental information facilitates performance gains, it does not fully resolve the reasoning challenges inherent in complex diagnoses. Even with access to comprehensive laboratory and imaging results, LLMs still exhibit systematic flaws in the final reasoning stage, most notably the neglect of negative evidence [36]. This suggests that diagnostic failures in rare disease scenarios are linked not only to information scarcity but also to difficulties in the logical processing of exclusionary findings. Therefore, simply increasing the volume of information input may be insufficient to overcome the underlying reasoning patterns that limit LLM performance in complex diagnostic tasks.

### Model-Specific Differences Under Diagnostic Complexity

Another important observation is that differences between models were relatively small in COPD but became more pronounced in RP. This suggests that model performance may converge under conditions where diagnostic patterns are clear and well-represented, but diverge when cases require handling uncertainty or integrating less typical information [41].

In relatively straightforward scenarios, most models are able to generate similar high-probability outputs, resulting in comparable performance. However, in more complex or ambiguous cases, models may differ in how they prioritize competing diagnostic possibilities or respond to incomplete information. These differences may reflect variations in training data composition, model architecture, or alignment strategies [42].

Qualitative observations provide preliminary insights into these model-specific patterns. In RP diagnosis, Leftdoctor GPT exhibited a higher frequency of errors, particularly in the neglect of negative evidence, which may indicate challenges in integrating exclusionary information. Kimi showed a broader distribution across error categories, whereas DeepSeek’s errors were more concentrated in feature identification and the neglect of negative evidence. In contrast, Doubao achieved the highest diagnostic accuracy in the RP group with the fewest total errors and no dominant error type, suggesting a relatively balanced performance across different reasoning domains. It is important to note that, given the limited number of error events in this qualitative analysis, these observations should be regarded as preliminary rather than definitive conclusions about model capabilities. Further validation in larger-scale studies is required to confirm these behavioral patterns.

### Clinical and Research Implications

The findings of this study suggest that the clinical use of LLMs may be context-dependent. While these models provide relatively reliable diagnostic support for common diseases with typical presentations, their outputs in rare or atypical cases require careful interpretation due to lower stability and higher intermodel variability.

From a clinical perspective, the identified error patterns offer practical guidance for diagnostic assistance. Clinicians should specifically verify whether AI-generated suggestions have appropriately integrated all exclusionary findings to ensure that sufficient diagnostic breadth is maintained for complex presentations. From a research perspective, these results underscore the importance of evaluation frameworks that reflect real-world, stepwise clinical reasoning. Such approaches may provide more realistic estimates of model performance compared to single-step evaluations based on complete information. Additionally, the preliminary error profiles identified in this study can guide targeted improvements in diagnostic capabilities. Future work could explore specialized prompting strategies or fine-tuning methods to help models weigh negative evidence and integrate multisystem clinical information more effectively. Beyond diagnostic reasoning, advancing Chinese LLMs will require expanding their applications across diverse medical specialties and complex decision-making scenarios. Integrating multimodal data such as medical imaging, pathology, and genetic information may help address the instability and information dependence observed in rare disease reasoning [43]. Specialty-specific optimization and cross-disease validation are critical for enhancing generalizability and reliability, while assessment frameworks that incorporate error patterns can provide a more comprehensive measure of clinical reasoning than accuracy metrics alone. Because demographic information such as age and sex was preserved in the clinical vignettes, the observed diagnostic performance may partially reflect how LLMs use demographic cues during diagnostic reasoning. Future studies should further investigate the extent to which demographic characteristics influence diagnostic outputs and whether demographic-related biases exist across different models.

### Limitations

This study has several limitations. First, the sample size was relatively small, leading to sparse-data instability in some statistical estimates, as reflected by large ORs with wide CIs. Although the sample size is consistent with prior exploratory studies of LLM diagnostic performance, larger multicenter studies are warranted to validate these findings across more diverse clinical scenarios [32]. Second, all models were tested using a standardized single prompt, which ensured fair comparisons but may not reflect their performance under alternative prompting strategies. In addition, each prompt was evaluated in a single run, which does not allow assessment of stochastic variability in model outputs; multiple runs could have better quantified or mitigated random fluctuations inherent to LLM-based systems. Third, our clinical vignettes were curated to exclude major comorbidities and included only confirmed cases with complete information. While we naturalized the language to reflect real patient narratives rather than textbook-style descriptions, this design may yield performance estimates that are higher than those observed in unselected real-world clinical practice. Fourth, while the vignettes were paraphrased, the use of public datasets for benchmarking involves an inherent, unquantified risk of semantic contamination. Since language models operate on semantic embeddings rather than relying solely on exact string matches, text modification may not entirely preclude the recognition of cases encountered during training. Finally, our model selection represents a snapshot based on benchmark rankings during the study period and does not encompass all representative Chinese LLMs. Given the rapid evolution of AI technology, performance rankings may shift as new models are released, and future research should incorporate a broader, more up-to-date range of models to provide a more comprehensive evaluation of their evolving capabilities.

### Conclusions

This study evaluated the diagnostic capabilities of 4 Chinese LLMs for common (COPD) and rare (RP) diseases using a step-by-step, clinical vignette-based approach. LLMs demonstrated strong diagnostic performance for common diseases but substantially lower and more variable performance for rare diseases. This performance disparity reflects the interplay between training data prevalence and the inherent challenges of synthesizing multisystemic clinical information. Although stepwise clinical information improved accuracy in rare disease cases, it did not eliminate the underlying reasoning limitations, particularly in integrating information and weighing evidence, which remained even when complete data were available. These findings suggest that current LLMs may perform well in pattern-consistent conditions but remain limited in handling diagnostic uncertainty and complexity. Careful evaluation and appropriate clinical oversight are therefore essential for their safe application in practice.

## Supplementary material

10.2196/89963 Multimedia Appendix 1 Evaluation framework and diagnostic performance analysis of large language models.

10.2196/89963 Multimedia Appendix 2 Sensitivity analysis results for generalized estimating equation (GEE) models.

10.2196/89963 Checklist 1 STARD 2015-Checklist.

## Abbreviations

CMCR: China Clinical Case Results Database
COPD: chronic obstructive pulmonary disease
DDx: differential diagnosis
GEE: generalized estimating equation
GOLD: Global Initiative for Chronic Obstructive Lung Disease
*ICD-10*: *International Classification of Diseases, Tenth Revision*
LLM: large language model
MCQ: multiple choice question
MRR: mean reciprocal rank
NLP: natural language processing
OR: odds ratio
RFA: rate of final diagnostic accuracy
RP: relapsing polychondritis
RTop3D: rate of final diagnosis within the top 3 DDx list
RTopD: rate of final diagnosis as top diagnosis
STARD: Standards for Reporting Diagnostic Accuracy Studies
