# Supplementary material for: Stepwise Diagnostic Evaluation of Chinese Large Language Models: Comparative Study of Common and Rare Diseases
Source: J Med Internet Res. 2026 Aug 6;28:e89963. doi: 10.2196/89963 (PMC13446800; doi:10.2196/89963)
Supplement: Supplementary file 1 [file jmir-v28-e89963-s001.doc]

**Section 1. Full Text of the Standardized Chinese Prompt with English Translation**

[Chinese Version - Original]

你是一名医生，你将：

1、根据我提供给你的病史，按诊断可能性的优先次序形成三个鉴别诊断，

每个鉴别诊断只能是一种疾病。

2、在你给出鉴别诊断后，我将提供给你进一步的检查报告。请基于你的鉴

别诊断，结合检查结果，给出最终诊断。

[English Translation]

You are a physician and you will:

1. Based on the medical history I have provided to you, form three differential diagnoses in order of diagnostic likelihood of prioritization, each of which can only be one disease.

2. After you have given your differential diagnoses, I will provide you with reports of further tests. Please give a final diagnosis based on your differential diagnosis, combined with the examination results.

**Table S1.** Diagnostic Capacity Outcome Indicator

| Indicator | Assessment method |
| --- | --- |
| The rate of final diagnosis within the top 3 DDx list (RTop3D) | Cases with correct diagnosis in top 3 differential diagnoses/Total cases*100% |
| The rate of final diagnosis as top diagnosis (RTopD) | Cases with correct diagnosis as the first differential diagnosis/Total cases*100% |
| The rate of final diagnostic accuracy (RFA) | Cases with correct final diagnosis/Total cases*100% |

**Table S2. MRR across LLMs by disease group (N = 28)**

| **Disease** | **LLM** | **Mean (SD)** | **Friedman χ²** | ***P-*values** |
| --- | --- | --- | --- | --- |
| COPD | — | — | 1.385 | .709 |
|  | Deepseek | .89 (.31) |  |  |
|  | KIMI | .89 (.31) |  |  |
|  | Doubao | .82 (.39) |  |  |
|  | Leftdoctor GPT | .89 (.31) |  |  |
| RP | — | — | 21.933 | <.001 |
|  | Deepseek | .37 (.46) |  |  |
|  | KIMI | .14 (.32) |  |  |
|  | Doubao | .39 (.48) |  |  |
|  | Leftdoctor GPT | .10 (.28) |  |  |

Note: Differences across the four models were assessed using the Friedman test based on case-level reciprocal rank scores, given the repeated-measures design across the same 28 clinical cases. Post-hoc pairwise comparisons were performed using Wilcoxon signed-rank tests with appropriate correction. MRR = Mean Reciprocal Rank; LLM = Large Language Model; SD = Standard Deviation; COPD: chronic obstructive pulmonary disease; RP: relapsing polychondritis.

**Table S3. Pairwise comparisons of MRR among LLMs for RP**

| **Comparison Pair** | ***Z*** | ***P-*values** |
| --- | --- | --- |
| DeepSeek vs KIMI | -2.537 | .011 |
| DeepSeek vs Doubao | -.408 | .683 |
| DeepSeek vs LeftDoctor GPT | -2.871 | .004 |
| Doubao vs KIMI | -2.668 | .008 |
| LeftDoctor GPT vs KIMI | -.816 | .414 |
| LeftDoctor GPT vs Doubao | -2.877 | .004 |

Note: Bonferroni correction was used for multiple pairwise comparisons. A *P*-value < .0083 was considered statistically significant. All pairwise comparisons were conducted at the case level using reciprocal rank values. MRR: mean reciprocal rank; LLMs: large language models; RP: relapsing polychondritis.

**Figure S1. Case Editing (Using COPD Cases as an Example)**
